# Intrastromal Corneal Ring Segment Implantation (Keraring 355°) in Patients with Central Keratoconus: 6-Month Follow-Up

**DOI:** 10.1155/2015/916385

**Published:** 2015-01-05

**Authors:** Khosrow Jadidi, Seyed Aliasghar Mosavi, Farhad Nejat, Mostafa Naderi, Leila Janani, Sara Serahati

**Affiliations:** ^1^Department of Ophthalmology, Bina Eye Hospital Research Center, Tehran 1914853184, Iran; ^2^Department of Epidemiology and Biostatistics, School of Public Health, Tehran University of Medical Sciences (TUMS), Tehran, Iran; ^3^Department of Biostatistics, University of Social Welfare and Rehabilitation Sciences, Tehran, Iran

## Abstract

We evaluate the efficacy and safety of Keraring 355° intrastromal corneal ring segment (ICRS) implantation aided by PocketMaker microkeratome for the correction of keratoconus. Patients underwent ICRS insertion using mechanical dissection with PocketMaker microkeratome and completed 6 months of follow-up. Uncorrected visual acuity (UCVA), best spectacle-corrected visual acuity (BSCVA), refraction, topographic findings, safety, efficacy index, and adverse events were reported for six months postoperatively. We evaluated 15 eyes of 15 patients (12 men) with a mean age of 28.87 ± 6.94 years (range 21–49 years). At final postoperative examination, there was a statistically significant reduction in the spherical equivalent refractive error compared to preoperative measurements (−5.46 ± 1.52 to −2.01 ± 1.63 D, *P* < 0.001). Mean preoperative UCVA (logMAR) before implantation was 0.79 ± 0.48, and postoperative UCVA was 0.28 ± 0.15, *P* = 0.001. Mean preoperative BSCVA (logMAR) before implantation was 0.36 ± 0.21; at final follow-up examination BSCVA was 0.18 ± 0.9, *P* = 0.009. Mean *K* decreased from 48.33 to 43.31 D, *P* < 0.001. All patients were satisfied with ICRS implantation; 86.7% were moderately to very happy with the results. No intraoperative or postoperative complications were demonstrated. This preliminary study shows that ICRS (Keraring 355°) implantation is an efficient, cost-effective, and minimally invasive procedure for improving visual acuity in nipple type keratoconic corneas.

## 1. Introduction

Keratoconus is a bilateral, progressive, noninflammatory disease of the cornea which often leads to high myopia and astigmatism with an estimated prevalence of approximately 1 in 2000 [1]. In the general population, the incidence of keratoconus is estimated to be between 50 and 230 per 100,000 [2–4]. It seems to be a multifactorial disease with an unknown exact etiology which impairs the quantity and quality of vision secondary to thinning in and protrusion of the cornea. This results in an irregular astigmatism with or without myopia [5–7]. Despite the fact that only one eye may be affected initially, keratoconus ultimately affects both eyes [8]. The conservative management of keratoconus in early stages consists of spectacle correction or rigid contact lenses. In more advanced stages with severe corneal irregular astigmatism and stromal opacities, surgical treatment with deep lamellar keratoplasty and penetrating keratoplasty (PK) should be considered [9–13].

Intrastromal corneal ring segments (ICRSs) represent a substantial evolution in the management of keratoconus. Moreover, long-term data on ICRS procedures demonstrated promising results in topographic regularity and uncorrected visual acuity (UCVA), indicating the “possibility of putting back or even replacing keratoplasty in keratoconus patients” [14, 15].

Different brands of ICRSs are currently on the market, including Intacs (Addition Technology, Inc.), Ferrara (Ferrara Ophthalmics Ltd.), and Keraring (Mediphacos Ltd.). Kerarings are made of medical grade polymethyl methacrylate (PMMA) with a UV blocker. They are characterized by a triangular cross section with variable thickness and an arc length that induces a flattening effect on the cornea. Keraring 355° intrastromal corneal ring (ICR; Mediphacos, Minas Gerais, Brazil) is a new unique intracorneal ring design especially developed for a nipple type keratoconus. It is available in a diameter of 5.7 mm and a thickness range of 200 and 300 *μ*m. To our knowledge, there are no reports on the effect of insertion or implantation of Keraring 355° on the postoperative outcome. To investigate the short-term visual and refractive outcomes after implantation of Keraring 355°, we conducted the current study in which all eyes had a 6-month follow-up.

## 2. Materials and Method

This prospective, consecutive, interventional study included 15 eyes from 15 patients (12 men, 3 women) with a mean age of 28.87 ± 6.94 years (range 21 to 49 years) with keratoconus. It was approved by The Institutional Review Board of the Eye Research Center, Bina Eye Hospital, and followed the tenets of the Declaration of Helsinki. After fully explaining the purpose and procedures of the study, all patients were asked to sign an informed consent form before treatment. Inclusion criteria were nipple type keratoconic eyes with clear central cornea, age between 21 and 49 years, minimum corneal thickness of 360 microns, mean keratometry between 45 and 52 D, contact lens intolerance, an uncorrected visual acuity (UCVA) not better than 20/50, and no visual dysfunctions other than keratoconus. Contact lens wear was discontinued three weeks prior to the exams. Exclusion criteria were positive pregnancy test, breast-feeding, history of vernal and atopic keratoconjunctivitis, history of keratorefractive surgery on the operative eye, patients with dry eye, history of corneal stromal disorders, nystagmus, immunosuppressive drugs users, hyperopia, advanced keratoconus with inferior corneal thinning less than 360 *μ*m, and patients with severe ocular and systemic pathologies (e.g., history of herpes keratitis, diagnosed autoimmune disease, systemic connective tissue disease, glaucoma, cataract, diabetic retinopathy, and age-related macular degeneration). A complete ophthalmic examination was performed preoperatively and postoperatively, including uncorrected visual acuity (UCVA), best spectacle-corrected visual acuity (BSCVA), manifest refraction, spherical equivalent (SE), keratometry (*K*) readings, and ultrasound pachymetry. Corneal topography was measured using the Orbscan II Slit Scanning Corneal Topography/Pachymetry System (Orbscan II, Bausch & Lomb). Visual acuity was measured using Snellen notation and then converted to logMAR for statistical analysis. Diagnosis of keratoconus was established by the combination of computerized video keratography of the anterior and posterior corneal surfaces (Orbscan IIz), *K* readings, and corneal pachymetry [16, 17]. The safety of implantation of Keraring 355° in patients with keratoconus was assessed using a refractive surgery safety index (safety index = postoperative best-corrected visual acuity ÷ preoperative best-corrected visual acuity). Efficacy was assessed using a refractive surgery efficacy index (efficacy index = postoperative uncorrected visual acuity ÷ preoperative best-corrected visual acuity) [18, 19].

Furthermore, we assessed patient satisfaction with three different questions. We asked every patient about their overall satisfaction with ICRS implantation after three years based on a six-point Likert scale ((0) no satisfaction; (1) very little satisfaction; (2) little satisfaction; (3) moderate satisfaction; (4) high satisfaction; (5) very high satisfaction). We also asked patients the questions “Would you recommend this procedure to other patients?” And “Would you have ICRS implantation for the other eye?”

### 2.1. Surgical Procedure

**Figure 1:**
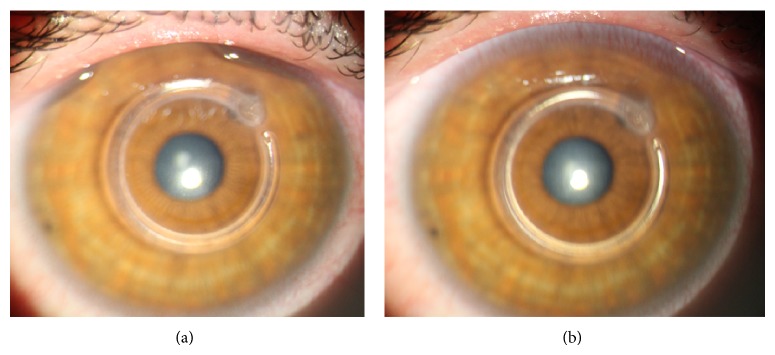
Slit-lamp examination of an eye with keratoconus one month and 3 months after Keraring 355° ICR implantation.

All surgical procedures were performed by the same experienced surgeon (Khosrow Jadidi) in an operating room under topical anesthesia with proparacaine hydrochloride 0.5% (Alcaine, Alcon) drops. In order to mark the central point of intrastromal corneal ring implantation, the operation microscope (OMS-800 Standard TOPCON Corporation, Japan) was used. In addition to the above, the pupil center was marked for proper centralization. The surgical procedure included creation of a pocket within the corneal stroma of 8.5 mm in diameter at 300-micron depth using a PocketMaker microkeratome (Dioptex GmbH) as described elsewhere [28, 38, 39] with a minor modification: when correct position of the blade was determined, the microvibrating diamond blade was set at 300 *μ*m of the measured corneal thickness and a single 2 mm radial incision was made at the steepest meridian. Then, the applicator was fixated to the eye by the suction ring. The suction ring was removed from the eye after creating a closed intrastromal pocket of 8.5 mm diameter and 300 *μ*m depth through the small incision tunnel. The Keraring 355° segment was inserted at the steepest meridian into the circular channel via the notch, using implantation forceps. The appropriate Keraring 355° segment thickness was selected and then implanted in the eye according to the new nomogram designed based on the author's experiences (Table 1 and Figure 1). The centration of the implant was adjusted using keratoscope. Subsequently, a silicone-hydrogel bandage contact lens (Bausch & Lomb) was placed on the cornea. Postoperatively, patients were prescribed betamethasone drops (Sina Darou) four times a day, chloramphenicol drops (Sina Darou) four times a day, and nonpreserved artificial tears (Artelac, Bausch & Lomb, France) six times per day. Chloramphenicol drops were discontinued one week postoperatively, but betamethasone drops were tapered after four to six weeks. The bandage contact lens was removed one day postoperatively. Patients were then scheduled for postoperative clinical examinations at one month and three and six months.

### 2.2. Statistical Analysis

Continuous variables with normal distribution are presented as mean ± SD. The paired *t*-test was used to compare preoperative and postoperative values of UCVA, BSCVA, SE, *K*_max⁡_, *K*_min⁡_, and *K*_mean_. The difference as a function of time was analyzed using paired two-tailed *t*-tests (at time intervals before operation to three months, before operation to six months, and three to six months of the follow-up period). Statistical analysis was performed using the SPSS 18.0 software (SPSS Inc., Chicago, IL, USA). *P* values less than 0.05 were considered statistically significant.

## 3. Results

**Figure 2:**
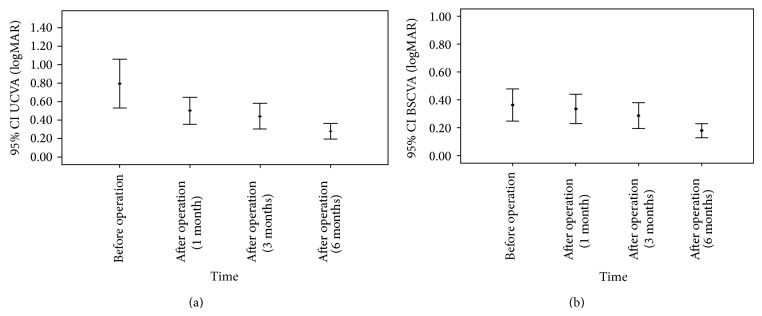
Mean change in visual acuity. Mean uncorrected visual acuity (a) and best spectacle-corrected visual acuity (b) after Keraring 355° implantation during the follow-up period.

**Figure 3:**
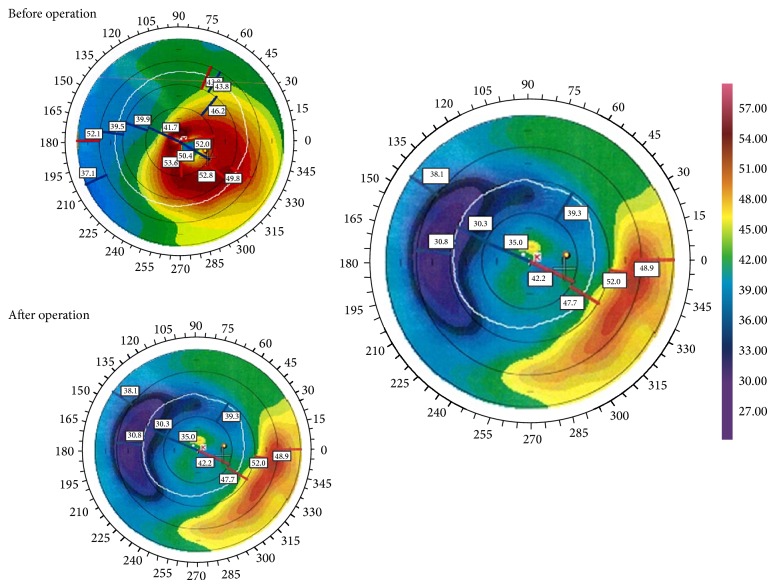
Preoperative (top left) and postoperative topographies at 3 months (top right) after Keraring 355° ICR implantation.

In this study, 15 eyes of 15 patients were evaluated. The mean age of the patients was 28.87 ± 6.94 years (range 21 to 49 years), and the male/female ratios were 4 : 1 (Table 2). The last postoperative follow-up time was six months. No intraoperative or postoperative complications were detected in this series of patients. Postoperatively, all eyes showed excellent corneal tolerance to the intrastromal corneal segments. The mean UCVA improved significantly from 0.79 ± 0.48 logMAR preoperatively to 0.28 ± 0.15 logMAR (*P* = 0.001) six months after implantation (Figure 2). The efficacy index was 3.12 at six months. The mean preoperative BSCVA was 0.36 ± 0.21 logMAR. The mean BSCVA improved to 0.18 ± 0.9 logMAR (*P* = 0.009) at six months after implantation (Figure 2). The safety index was 1.26 at six months. There was a significant improvement in spherical equivalent refractive error from −5.46 ± 1.52 diopters (D) preoperatively to −2.01 ± 1.63 (*P* < 0.001) at 6 months postoperatively (Table 3). The mean *K* readings improved in the same period, from 48.11 ± 1.95 D to 43.31 ± 2.31 (*P* < 0.001) (Figure 3). Our results present a significant flattening effect postoperatively, since the mean topographic *K* values showed decreases in *K*_mean_, *K*_max⁡_, and *K*_min⁡_ at six months postoperatively (*P* < 0.001). The means (standard deviation) of all data are shown in Tables 3 and 4. Postoperatively, UCVA and BSCVA showed a significant improvement and sphere, cylinder, SE, and keratometry readings were significantly reduced. Moreover, postoperatively, all eyes showed excellent corneal tolerance to intrastromal corneal segment. In addition, all patients were satisfied with Keraring 355° implantation. Likewise, on a scale of 0 to 5 for current overall satisfaction, 86.7% of patients noted that they were moderately to very happy with the results (scores 3–5) (Table 5).

## 4. Discussion

The purpose of the present study was to determine the effects of Keraring 355° on uncorrected visual acuity (UCVA), best spectacle-corrected visual acuity (BSCVA), refraction, topography, and the safety and efficacy indices in keratoconic eyes.

Several possible alternatives to manage keratoconus have been reported in the literature, including scleral-fitted gas-permeable contact lenses, inferior eccentric penetrating grafts, deep lamellar keratoplasty, penetrating keratoplasty, and a recently developed therapeutic tool: intrastromal corneal ring segments (ICRSs).

ICRSs were designed with the goal of delaying or avoiding corneal grafts in keratoconus patients. It represents a prominent evolution in the management of keratoconus via flattening the central corneal curvature to achieve a refractive adjustment due to the removable and tissue saving nature of the technique. The high efficiency of Intacs in correcting keratoconic eyes has been reported by several authors [9–14, 23–30]. ICRS implantation in post-Lasik ectasia appears to be safe and effective in decreasing myopia, corneal steepness, and decentration of the corneal apex and offers potential improvement of UCVA and BSCVA in keratoconus patients [9, 20, 22, 24, 25, 30–40].

Keraring 355° is a new intracorneal ring design made of polymethyl methacrylate (PMMA) and is especially developed for a nipple type keratoconus. Femtosecond laser is the suggested technique for implantation. However, manual and mechanical techniques are not prohibited.

Mechanical dissection and femtosecond laser are the two main techniques generally used for tunnel creation during ICRS implantation [41]. The traditional mechanical technique for tunnel creation can cause complications such as epithelial defects at the keratotomy site, extension of the incision, anterior and posterior perforations, infectious keratitis, shallow placement of intrastromal corneal ring segments, decentration, asymmetric placement, persistent incisional gaping, corneal stromal edema around the incision, and stromal thinning [21, 22, 32].

A study by Hellstedt et al. demonstrated a 35% rate of postoperative complications such as corneal melt, segment movement, and exposure with the mechanical tunnel dissection method [32]. These complications could be reduced with femtosecond laser due to the more precise localization, dimensions, diameter, depth, and width of the channel. Despite this, Ferrer et al. found no significant difference between the use of femtosecond laser and mechanical dissection [42]. Also, a significant improvement in CDVA, UDVA, and *K* readings after ICRS implantation was reported with a mechanical and femtosecond laser tunnel creation in other studies [43–45].

Coskunseven et al. reported improvement of CDVA in 15.68% of 50 eyes, a decrease in the mean keratometry from 50.6 D to 47.5 D and the mean SE from −5.6 D to −2.4 D after ICRS implantation at 1 year [43]. Similarly, Kubaloglu et al. compared the outcomes of Keraring ICRS implantation with mechanical and femtosecond laser tunnel creation and demonstrated an improvement of the UDVA and CDVA in 86% and 88% of eyes, respectively. In addition, a decrease in the mean maximum *K* value from 53.5 D to 48.9 D and an improvement in the mean SE from −5.05 to −1.87 D at 1 year in femtosecond laser group were found. The UDVA and CDVA were improved in 88% and 84% of eyes, respectively. The mean maximum *K* value decreased from 54.1 D to 43.8 D, and the mean SE went from −5.75 to 0.75 D at 1 year in the mechanical group [44].

In our study, we used the mechanical technique to create a pocket within the corneal stroma with the PocketMaker microkeratome (Dioptex GmbH). According to our experiences, this technique is substantially less expensive than the femtosecond laser technique. Secondly, decentralization is manageable and even reversible. Thirdly, with the mechanical technique, reshaping and remodeling of the cornea are more feasible than with the femtosecond laser technique.

In our cases, postoperative results revealed a significant reduction in the magnitude of corneal steepening, an increase in topographical regularity, and an improvement in the UCVA and BSCVA when the Keraring 355° was implanted at 300 *μ*m thickness (see Table 3). Furthermore, all operations were uneventful, and no extrusions of the rings were found. Additionally, the integrity of the cornea was well preserved in all eyes.

On one hand our results are in contrast to the study by Kwitko and Severo [33] that demonstrated a higher rate of extrusion using standard mechanical stromal dissection for Keraring implantation. On the other hand, despite the small sample of eyes (15 eyes) in our study, our finding is similar to the study by Shabayek and Alió [45] using femtosecond laser for keratoconus correction. They found a significantly increased UCVA from 0.06 to 0.3 and BSCVA from 0.54 to 0.7, and the spherical equivalent and the average keratometric values (*K* value) decreased by 2.28 diopters (D) and 2.24 D, respectively. The major changes in refraction and topographic findings in our series seem to take place during the early postoperative period (the first three postoperative months) for UCVA but BSCVA improved after this period, contradicting the results of Shabayek and Alió [45] which showed no significant difference between the 3- and 6-month follow-ups.

These results were similar to the results of Intacs implantation in low myopia patients [46–48], patients with keratoconus [9, 23–27], and patients with post-LASIK ectasia [14, 24, 29] where stability in refraction and visual acuity after the sixth month were observed.

Additionally, in our study, the efficacy index and the safety index were more than 1.00 at six months postoperatively, which showed the visual outcomes were satisfactory. Additionally, all patients were satisfied with Keraring 355° implantation and the majority of cases agreed to have an implant inserted in the other eye (data not shown). We believe that this new technique with the unique and specialized characteristics of the Keraring 355° may explain the reliable results in our study.

Our study has potential limitations, including the small sample of treated eyes, the lack of higher-order aberration analysis, and the lack of a control group. However, the results in our study are similar to those in a keratoconus study in which ICRSs were used for treatment [43–45].

In conclusion, we have shown that ICRS (Keraring 355°) implantation using a mechanical dissection with the PocketMaker microkeratome is a unique, safe, efficient, and minimally invasive procedure in treating nipple type keratoconic eyes, and it reduces the risk of operative and postoperative complications. Further studies with a longer follow-up period and a larger number of patients are recommended to draw final conclusions about the efficacy and safety of ICRSs (Keraring 355°) and their role in controlling the progression of keratoconus.

This study is underway, and the results will be reported soon.

## Figures and Tables

**Table 1:** Keraring 355° ICR nomogram.

Spherical equivalent	Keraring 355° ICR dimension	
	Diameter (mm)	Thickness
<6 D	5.7 mm	200 μm
>6 D	5.7 mm	300 μm

ICR: intrastromal corneal ring.

**Table 2:** Characteristic of participants.

Number of patients	15
Number of eyes	
OD (%)	8 (53.3%)
OS (%)	7 (46.7%)
Sex	
Male (%)	12 (80%)
Female (%)	3 (20%)
Age	
Mean (SD)	26.06 (3.67)
Range	21–35

**Table 3:** Comparison between preoperative and postoperative visual outcomes.

	Preoperative	1-month postoperation	3-month postoperation	6-month postoperation	*P* value 3 versus pre.	*P* value 6 versus pre.	*P* value 6 versus 3
*UCVA* *(logMAR) *							
Mean (SD)	0.79 (0.48)	0.50 (0.26)	0.44 (0.25)	0.28 (0.15)	0.005^**^	0.001^**^	0.002^**^
*BSCVA* *(logMAR) *							
Mean (SD)	0.36 (0.21)	0.34 (0.19)	0.29 (0.17)	0.18 (0.09)	0.19	0.009^**^	0.007^**^
*Sphere (D) *							
Mean (SD)	−2.38 (1.85)	−0.33 (2.34)	−0.62 (2.79)	−0.25 (2.27)	0.052	0.019^*^	0.242
*Cylinder (D) *							
Mean (SD)	−4.27 (1.25)	−2.18 (0.82)	−1.78 (1.22)	−1.88 (0.95)	<0.001^**^	<0.001^**^	0.714
*SE (D) *							
Mean (SD)	−5.46 (1.52)	−2.35 (1.67)	−2.09 (2.19)	−2.01 (1.63)	<0.001^**^	<0.001^**^	0.822

Notes: UCVA: uncorrected visual acuity; BSCVA: best spectacle-corrected visual acuity; D: diopters; logMAR, logarithm of the minimum angle of resolution; SD: standard deviation; SE: spherical equivalent. Significances are based on paired *t*-test. ^*^ *P* < 0.05; ^**^ *P* < 0.01.

**Table 4:** Comparison between preoperative and 6-month postoperative *K* values.

	Preoperative	6-month postoperation	*P* value
*K*_max⁡_ value (D)			
Mean (SD)	50.39 (2.14)	44.22 (2.17)	<0.001^**^
*K*_min⁡_ value (D)			
Mean (SD)	45.85 (1.94)	42.14 (2.53)	<0.001^**^
*K*_mean_ value (D)			
Mean (SD)	48.11 (1.95)	43.31 (2.31)	<0.001^**^

Note: SD: standard deviation; D: diopter; significances are based on paired *t*-test. ^**^ *P* < 0.01. *K*_min⁡_: minimum curvature; *K*_max⁡_: maximum curvature; *K*_mean_: mean curvature.

**Table 5:** General satisfaction of participants 6 months after operation.

Satisfaction score (*N* = 15)	Frequency (%)	Mean (SD)
No	0.0 (0.0%)	4.27 (1.16)
Very little	2.0 (13.3%)	
Little	0.0 (0.0%)	
Moderate	7.0 (46.7%)	
Much	4.0 (26.7%)	
Very much	2.0 (13.3%)	
